# Protocol for hybrid mass spectrometry-AI mapping of proteasome-generated HLA class I epitopes

**DOI:** 10.1016/j.xpro.2025.104154

**Published:** 2025-10-22

**Authors:** Maxim T. Ri, George A. Saratov, Anna A. Kudriaeva, Alexey A. Belogurov

**Affiliations:** 1 Center for Molecular and Cellular Biology, Moscow 121205, Russia; 2 Shemyakin and Ovchinnikov Institute of Bioorganic Chemistry, Russian Academy of Sciences, Moscow 117997, Russia; 3 Department of Biological Chemistry, Russian University of Medicine, Ministry of Health of Russian Federation, Moscow 127473, Russia

**Keywords:** Sequence analysis, Immunology, Protein expression and purification, Mass Spectrometry, Systems biology

## Abstract

Proteasomes play a central role in antiviral and antitumor cytotoxic immunity, generating most of the >10,000 unique proteome-derived peptides bound to ∼200,000 human leukocyte antigen (HLA) class I molecules on each human cell. Here, we present a protocol for hybrid mass spectrometry-AI mapping of proteasome-generated HLA class I epitopes. We describe steps for *in vitro* processing of antigens by proteasomes, HLA class I allele selection, and peptide pool preparation. We then detail procedures for determining HLA class I ligands and population immunity.

For complete details on the use and execution of this protocol, please refer to Kudriaeva et al.^1^

## Highlights

• Steps for obtaining cell lines stably expressing HTBH-tagged proteasomes
• Instructions for *in vitro* processing of antigens by proteasomes
• Guidance on applying artificial AI to predict potential HLA class I peptide binders
• Procedures for identifying physiologically relevant pools of HLA class I epitopes

## Abstract (editor highlights)

Publisher’s note: Undertaking any experimental protocol requires adherence to local institutional guidelines for laboratory safety and ethics.

## Graphical abstract

**Figure:**
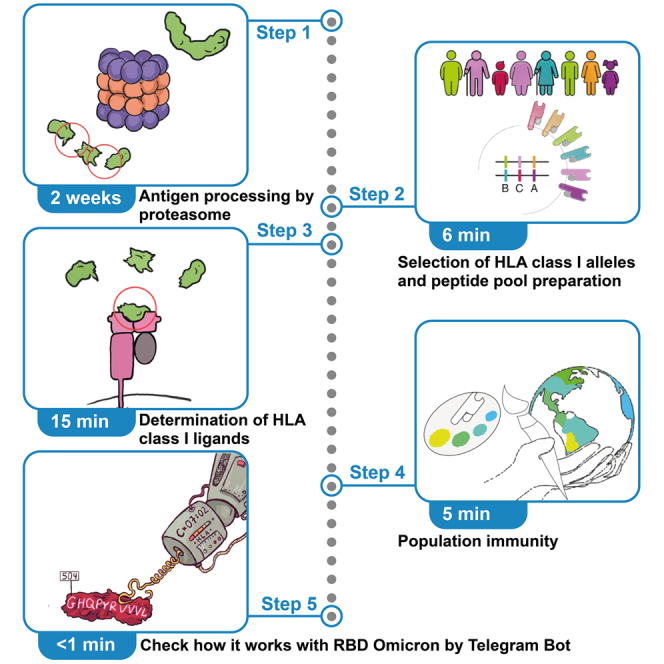

## Before you begin

This protocol describes the procedure used to fragmentate antigens by proteasomes with different catalytic phenotypes. It demonstrates how to utilize the Allele Frequency Net Database, which covers the global population haplotypes, ensuring the inclusion of diverse HLA allele distributions across ethnic groups. Protocol depicts peptide binding predictions using netMHCpan-4.1, an artificial neural network trained on extensive datasets of experimentally validated HLA-peptide binding affinities. This approach is particularly valuable for investigation of viral and self-antigens HLA class I-dependent presentation, as it allows rapid identification of potentially immunogenic peptides and their protective or pathogenic potential across different population groups. Below, we provide a step-by-step guide to this protocol, covering everything from preparation and data acquisition to analysis and post-analysis.

### Innovation

Multicatalytic proteinase complexes or proteasomes are an important element of adaptive immunity as peptide pools coming from proteasomes during protein hydrolysis are further presented on the cell surface in the context of the HLA class I molecules. In humans almost each cell except mature red blood cells contains around 200,000 HLA I bound with more than 10,000 unique proteome-derived peptides thus forming “molecular passport” – a signature scanned by T cell receptors, which is a basis for antiviral and antitumor CD8^+^ immunity. Numerous studies describe such epitopes, however majority of them operate with T cell assays utilizing overlapping synthetic peptides or *in silico* prediction algorithms. Thus, proteasome activity, which is responsible for generation of majority of HLA class I-exposed peptides, is beyond the resulting conclusions. As a result, this may significantly misrepresent interpretation of such datasets. Despite its *in vitro* activity in form of short peptides, majority of reported epitopes may not physically exist: not released or internally cleaved by proteasome during intracellular antigen processing, and thus has no any physiological meaning. Here we report more accurate way to predict pools of physiologically relevant and potentially immunodominant peptides by *in vitro* processing of proteins by proteasome together with subsequent analysis of its affinity to HLA class I molecules.

## Key resources table

**Table:** 

REAGENT or RESOURCE	SOURCE	IDENTIFIER
**Chemicals, peptides, and recombinant proteins**		

DMEM	Gibco	Cat# 12491023
PBS 1×, pH 7.4	Gibco	Cat# 10010023
Trypsin-EDTA 0.05%	Gibco	Cat# 25300054
“Cryomed-M″ medium	PanEco	Cat# С881-МЕ
Fetal bovine serum	Gibco	Cat# 10099141
Antibiotic-Antimycotic	Gibco	Cat# 15240062
GlutaMAX	Gibco	Cat# 35050061
Lipofectamine 3000	Life Technologies	Cat# 3000015
Puromycin	Gibco	Cat# A1113803
Tris	Molekula	Cat# 11942384
NaCl	Sigma-Aldrich	Cat# S9888
MgCl_2_	Amresco	Cat# Am-O288–0.1
TCEP	Sigma-Aldrich	Cat# C4706
Nonidet Р-40	Amresco	Cat# Am-E109–0.05
Acetonitrile	Concord Technology	Cat# 8002GG2500
Trifluoracetic acid	Sigma-Aldrich	Cat# T6508
Formic acid	Sigma-Aldrich	Cat# 1.00263
IFNγ human	Sigma-Aldrich	Cat# I3265
TEV protease, His	GenScript Biotech	Cat# Z03030
ProteinSafe Protease Inhibitor Cocktail	TransGen Biotech	Cat# DI111-01

**Experimental models: Cell lines**		

HEK 293T	VCCC	N/A
HeLa	VCCC	N/A

**Recombinant DNA**		

pSBi-PSMB4-HTBH-Puro	generated in this study	N/A
pCMV(CAT) T7-SB100	Addgene	Cat# 34879

**Software and algorithms**		

FragPipe 21.0	Kong et al.^2^	https://fragpipe.nesvilab.org/
MaxQuant 2.3.1.0	Cox et al.^3^	https://www.maxquant.org/
netMHCpan-4.1	N/A	https://services.healthtech.dtu.dk/services/NetMHCpan-4.1a/

**Other**		

Streptavidin agarose resin	Thermo Fisher scientific	Cat# 20353
PepMap Neo Trap Cartridge	Thermo Fisher scientific	Cat# 174500
Peaky Reprosil Pur C18 AQ	Molecta	Cat# Peaky-75-30
Cell culture flask 75 cm^2^ (T75) filter cap, treated, sterile	SPL	Cat# 70075
Superose 6	Cytiva	Cat# 29091597
Cryogenic vial 2.0 mL	NEST	Cat# 607001
Safe-Lock tubes 1.5 mL	Eppendorf	Cat# 0030120086
Safe-Lock tubes 2 mL	Eppendorf	Cat# 0030120094
6-Well flat bottom cell culture plate	SPL	Cat# 30006
Orbitrap Exploris 480 Mass spectrometer	Thermo Fisher scientific	Cat# BRE725539
Centrifuge 5425 R	Eppendorf	Cat# 5406000119
Multi-bio RS24	BioSan	Cat# BS-010117

## Materials and equipment

**Table:** Lysis buffer

Reagent	Final concentration	Amount
Tris-HCl (pH 7.5) (1 M)	30 mM	300 μl
MgCl_2_ (1 M)	5 mM	50 μl
Nonidet Р-40	0.5%	50 μl
TCEP (500 mM)	1 mM	20 μl
ProteinSafe Protease Inhibitor Cocktail	1×	100 μl
Total	N/A	10 ml with ddH2O

Prepare fresh, do not store.

**Table:** Wash buffer 1

Reagent	Final concentration	Amount
Tris-HCl (pH 7.5) (1 M)	30 mM	300 μl
MgCl_2_ (1 M)	5 mM	50 μl
NaCl (5 M)	750 mM	1.5 ml
TCEP (500 mM)	1 mM	20 μl
Total	N/A	10 ml with ddH2O

Store at 4C for up to 1 month.

**Table:** Wash buffer 2

Reagent	Final concentration	Amount
Tris-HCl (pH 7.5) (1 M)	30 mM	300 μl
MgCl_2_ (1 M)	5 mM	50 μl
TCEP (500 mM)	1 mM	20 μl
Total	N/A	10 ml with ddH2O

Store at 4C for up to 1 month.

**Table:** Loading solution

Reagent	Final concentration	Amount
Acetonitrile	5%	2.5 ml
Trifluoracetic acid	0.1%	50 μl
Total	N/A	50 ml with ddH2O

Prepare fresh, do not store.

**Table:** Solution A

Reagent	Final concentration	Amount
Formic acid	0.1%	50 μl
Total	N/A	50 ml with ddH2O

Prepare fresh, do not store.

**Table:** Solution B

Reagent	Final concentration	Amount
Acetonitrile	80%	40 ml
Formic acid	0.1%	50 μl
Total	N/A	50 ml with ddH2O

Prepare fresh, do not store.

**Table:** Full DMEM medium

Reagent	Final concentration	Amount
DMEM	N/A	440 ml
Fetal bovine serum	10%	50 ml
GlutaMAX	1×	5 ml
Antibiotic-antimycotic	1%	5 ml

Store at 4C for up to 6 months.

## Step-by-step method details

### Obtaining cell lines stably expressing HTBH-tagged proteasomes

**Timing: 11 days**

This step allows the creation of HEK 293T (for constitutive 20S proteasomes purification) and HeLa (for constitutive or immune 20S proteasomes purification) cell lines that stably express 20S proteasome subunit PSMB4 with the HTBH tag.

1. Grow HEK 293T or HeLa cells in full DMEM medium to 70% confluence in a 6 well plate.
2. Co-transfect cells in each well with 2 μg of Sleeping Beauty transposon plasmid pSBi-PSMB4-HTBH-Puro (Data S1) and 0.2 μg of Sleeping Beauty transposase plasmid pCMV (CAT) T7-SB100 with Lipofectamine 3000 according to manufacturer’s instructions.
3. Three days after transfection, maintain cells in selection medium (1 μg/mL puromycin in DMEM growth medium) for at least seven days.

***Note:*** After selection, it is recommended to freeze the cells. To do this, grow the cells to 90% confluence in a selective medium in T75 flask, rinse with 3 ml of 1× PBS, detach the cells using 3 ml of 0.05% trypsin-EDTA solution and resuspend in a full DMEM medium. Then, centrifuge the cells at 125 g for 10 min, discard the supernatant and resuspend cells in Cryomed-M at a ratio of 1 million cells per 1 ml. Freeze cells in cryogenic vials in liquid nitrogen.

### Purification of HTBH-tagged 20S proteasomes

**Timing: 2 days**

This step makes it possible to obtain a highly purified proteasome preparation from mammalian cells.

4. Grow stable eukaryotic cells expressing HTBH-tagged proteasomes in full DMEM medium to 90% confluence in T75 flask.

**CRITICAL:** Expose the HeLa cells to 500 U/mL of IFNγ for 72 h to induce expression of the proteasome catalytic immunosubunits directly after cell seeding.

5. Lyse the cells.
  a. Wash cells with 3 ml of PBS buffer to remove the residual growth medium.
  b. Add 1 ml of the lysis buffer into the T75 flask.
  c. Incubate cells in lysis buffer until they have detached from the plate (approximately 10 min).
  d. Collect the cell pellets into sterile 2 ml Eppendorf.
  e. Centrifuge the lysates at 20000 g, 4°C for 15 min to remove cellular debris.
  f. Transfer the supernatant to the new sterile 1.5 ml Eppendorf.
6. Purify biotinylated proteasomes.
  a. Wash 20 μL of streptavidin agarose resin three times by centrifugation at 500 g for 3 min with 1 ml of wash buffer 2.
  b. Resuspend the resin in 100 μl of Wash buffer 2.
  c. Add the resin to the supernatant obtained in step 2.
  d. Incubate the resin with the supernatant for 16 h at 4°C with constant rotation at 10 rpm.
  e. Wash the resin twice by centrifugation at 500 g for 3 min with 1 ml of wash buffer 1 (750 mM NaCl allows to separate the 19S subparticles from the 20S catalytic core).
    **CRITICAL:** Incubate the resin in wash buffer 1 for 2 min before centrifugation.
  f. Wash the resin twice by centrifugation at 500 g for 3 min with 1 ml of wash buffer 2.
  g. Discard the supernatant and resuspend the resin in 20 μl of wash buffer 2, containing 1 μl of TEV protease.
  h. Incubate the resin at 30°C for 2 h.
  i. Collect the supernatant with purified proteasomes.
  j. Optionally eluted proteasomes may be additionally purified by size-exclusion chromatography on Superose 6 (Cytiva, Marlborough, MA, USA) column in 20 mM Tris (pH 7.5), 5 mM MgCl_2_, and 1 mM TCEP.
    ***Note:*** The expected yield of a constitutive 20S and immune 20S proteasomes from one T75 flask is 20 μg and 5 μg respectively.

### Antigen hydrolysis by purified proteasomes

**Timing: 16 h**

This step allows you to process antigen into peptides by immune and constitutive 20S proteasomes.

7. Mix 1 μg of the 20S proteasomes obtained in the last step and 1.5 μg of antigen in 20 μl of wash buffer 2.
8. Incubate the mixture for 16 h at 37°C.

***Note:*** Incubate control antigen samples in same conditions without proteasomes. Not all antigens can be hydrolyzed equally effective. It may be necessary to preheat the protein at 95°С for 10 min to denature it. Avoid usage of detergents and chaotropic agents.

### LC-MS/MS analysis

**Timing: 3 h**

This step allows the peptides to be extracted from the solution and analyzed by the LC-MS/MS.

9. Dilute 1 μL of each sample with 10 μL of loading solution.
10. Inject 5 μL in trap-elute manner on trap column cartridge with 10 μL/min flow rate of loading solution.
11. Separate the peptides on capillary column.
12. Elute the peptides with mobile phase gradient from 5% of solution B in solution A at flow rate of 250 nL/min to 50% of solvent B in 10 min.
13. Detect the peptides in data-dependent acquisition mode on high-resolution quadrupole-orbitrap tandem mass-spectrometer Exploris 480.
  a. Set the electrospray ionization voltage at 2,200 V.
  b. Perform the precursor scan for ions wit m/z from 350 to 1400 at resolution 60 000 (at 200 m/z).
  c. Subject up to 30 precursors with charge from 2 to 6 to fragment ion scan (resolution 15 000 at 200 m/z).
  d. Set the normalized collision energy to 30%.

### LC-MS/MS data processing

**Timing: 1 h**

This step allows the identification of antigen peptides generated by proteasomes.

14. Perform the peptide identification with FragPipe software.
  a. Perform the search against a database containing sequences of chosen antigen, proteasome proteins and common contaminants.
  b. Use the non-specific cleavage.
  c. Consider peptides with mass from 500 to 12000 kDa and length from 7 to 65 residues.
  d. Set mass tolerance to 20 ppm with further optimization.
  e. Use oxidation of methionines, deamidation of asparagine and glutamine, N-terminal pyro-glutamine, N-term acetylation and serine, threonine and tyrosine phosphorylation formation as variable modifications.
15. Set FDR levels to 1% and perform validation with PeptideProphet and ProteinPropher.
16. Perform label-free quantification with MaxQuant software against a database of peptides identified with FragPipe with no cleavage at all with the same set of variable modifications.
17. Set FDR levels at 1% for PSM and protein levels.
18. Enable label-free quantification and matching between runs.

***Note:*** Median peptide length is routinely 13 amino acids (i.e., 8–16 aa range approximately 60% (6%–8% for each length with reduction to less than 3% for 17 and more amino acids), 8–10 aa – 20%, 75% of peptides are observed as 2+ and 3+ charged ions, and 1% FDR value results in 10%–15% peptide cutoff.

### Loading scripts and files

**Timing: 10 min**

This step downloads customs scripts and files from GitHub that are required for data processing on a Linux server. The Linux server must have gawk installed for the scripts to work properly. Additionally, please download **HLA haplotypes** and **netMHCpan-4.1**.^4^

19. Run the following command in the terminal:

git clone https://github.com/RiMaxim/Coronavirus.git

cd Coronavirus

chmod +x ∗sh ∗py

***Note:*** The file **input.csv** includes peptides generated by proteasomal cleavage of Omicron B.1.1.529 and Wuhan-Hu-1 receptor binding domain (RBD) protein sequences with HeLa c20S (constitutive proteasome), HeLa i20S (immunoproteasome), and a non-cleaved control sample.

Input file (input.csv):

YADSFVLRGDEVRQL,Omicron B.1.1.529,control,0.00001538417226173

ADSFVLR,Omicron B.1.1.529,HeLa c20S,0.00006347479612358

VLRGDEVRQLAPGQTGNLADYNY,Omicron B.1.1.529,HeLa i20S,0.00008557498424691

QGGTGLNDLFEAQKLEWHETGHHHHHH,Wuhan-Hu-1,control,0.00003667933530789

ADSFVLRGDEVRQL,Wuhan-Hu-1,HeLa c20S,0.00008092433737121

***Note:*** Some rows of **input.csv** file. The first column contains peptide sequence, second column – RBD type, third column – proteasome type (HeLa c20S, HeLa i20S) or control, fourth column – the ratio of peptide intensity to total ion current in the distinct sample.

20. It is necessary to prepare a file with a table from the website **Allele Frequency Net Database** specifically from the **BrowseGenotype.aspx** page.^5^
  a. You are in the “Coronavirus” folder, just like in the previous step.
  b. Open a text editor of your choice (e.g., nano, vim, or any graphical text editor).
  c. Copy the table without header and paste it into the editor.
  d. Save the file as **table.txt** in the desired directory.
  e. Run the following command in the terminal:

./download.sh

***Note:*** To automate the process, a bash shell script is used, which substitutes each ID into the URL and makes a request to the web resource using the wget command. All the obtained data are saved in a single file (**data.csv**). The file data.csv contains HLA haplotypes for 86,572 individuals (March 2025). The quantity may vary depending on the upload date. Each row represents a set of allele pairs for 7 loci (A, B, C, DRB1, DQB1, DPA1, and DPB1) for an individual.

Output file (data.csv):

1,A∗02,A∗68,B∗18,B∗51,C∗02,C∗14,DRB1∗11,DRB1∗16,DQB1∗03,DQB1∗05

2,A∗01,A∗32,B∗37,B∗44,untyped,untyped,DRB1∗11,DRB1∗11,DQB1∗03,DQB1∗03

3,A∗01,A∗02,B∗44,B∗52,C∗02,C∗12,DRB1∗14,DRB1∗15,DQB1∗05,DQB1∗06

4,A∗01,A∗02,B∗40,B∗40,C∗15,C∗15,DRB1∗14,DRB1∗14,DQB1∗05,DQB1∗05

21. On the website https://services.healthtech.dtu.dk/services/NetMHCpan-4.1/ fill out a form providing an email address from a university or academic institution.
22. Download the program in a compressed archive format using provided link. After downloading to the server (folder Coronavirus), unpacking and configuration are required.
23. Run the following command in the terminal:

tar -xvzf netMHCpan-4.1b.Linux.tar.gz

cd netMHCpan-4.1

wget https://services.healthtech.dtu.dk/services/NetMHCpan-4.1/data.tar.gz

tar -xvzf data.tar.gz

sudo apt install tcsh

***Note:*** The line 14 of the file netMHCpan, replace the path to the folder netMHCpan-4.1. If you do not have root privileges but need to use tcsh, for example, to run netMHCpan, you can install tcsh locally and modify the shebang in the scripts to point to your local copy.

### Processing file with antigen peptides

**Timing: <1 min**

24. The obtained file (**input.csv**) is processed and filtered using a bash script.
  a. To improve data reproducibility, aggregate intensity values by averaging replicates of the same peptide under equal experimental conditions (technical repeats).
    Input file (input.csv):
    VLSFELLHA,Omicron B.1.1.529,HeLa c20S,0.00008837341900559
    VLSFELLHA,Omicron B.1.1.529,HeLa c20S,0.00002438736699410
    Output file:
    VLSFELLHA,Omicron B.1.1.529,HeLa c20S,0.000056380393
  b. Create a unique list of peptides representing the antigen and proteasome type.
  c. For each group, calculate the average intensity, considering HeLa c20S and HeLa i20S as one group, while the antigen type is considered separately.
  d. Calculate the sum of the intensities for the control.
  e. The result is presented in a table, where the rows correspond to unique peptides and the columns represent unique combinations of antigen and proteasome type.
    Input file:
    ADSFVLRGDEVRQLAPGQ,Omicron B.1.1.529,HeLa c20S,0.00001972037238
    ADSFVLRGDEVRQLAPGQ,Omicron B.1.1.529,HeLa i20S,0.00004256270991
    ADSFVLRGDEVRQLAPGQ,Omicron B.1.1.529,control,0.00003221155346
    ADSFVLRGDEVRQLAPGQ,Wuhan-Hu-1,HeLa c20S,0.00003393182328
    ADSFVLRGDEVRQLAPGQ,Wuhan-Hu-1,HeLa i20S,0.00076722748889
    Output file:
    Peptide,Wuhan-Hu-1,Omicron B.1.1.529,Total value for the control group
    ADSFVLRGDEVRQLAPGQ,0.00040057965608,0.00003114154114,0.00003221155346
  f. Keep only those peptides for which the control intensity value equals to 0.
  g. In all peptides replace leucine residues (L) with isoleucine (I) or vice versa if the peptide did not align exactly to the antigen sequence.
  h. Withdraw peptides, which overlapped with non-native sequences, e.g. tags, linkers, fusion proteins. Run the following command in the terminal:
    cd ../
    ./preprocess_peptide.sh
    ***Note:*** The script processes all steps (24 a–h).
    Output file (peptide.csv):
    QPTNGVGY,498,505,0.00006275172738,0,Wuhan-Hu-1
    NYKLPDDF,422,429,0.00012261736936,0,Wuhan-Hu-1 and Omicron B.1.1.529
    VGHQPYRV,503,510,0,0.00000431685789,Omicron B.1.1.529
    ***Note:*** The output file (**peptide.csv**) is provided in CSV format. The first column contains the peptide sequence, the second column indicates the peptide start position within the antigen sequence, the third column is the end position, the fourth column shows the intensity of the peptide derived from the antigen 1, the fifth column shows the intensity of the peptide derived from the antigen 2, and the sixth column indicates the type of the antigen from which the peptide was derived.

### Processing of HLA haplotypes

**Timing: <1 min**

The obtained file (**data.csv**) is processed and filtered using a bash script.

25. It is necessary to standardize file to a unified format. Run the following command in the terminal:

./processing_HLA.sh data.csv data2.csv

***Note:*** The script performs the following steps: (1) The input **data.csv** is split by a comma (,) as the main column delimiter; (2) in each cell of the column containing separation by the “/” character, the first allele is selected. A regular expression is used to extract alleles matching the format A∗XX:YY, B∗XX:YY, and C∗XX:YY (HLA class I); (3) the alleles are sorted into three separate groups: A, B, and C; (4) alleles within each group are sorted in ascending order; (5) the groups are combined in the order A, B, C, removing empty values and unnecessary delimiters. The output file name is **data2.csv**. The file **data2.csv** contains 21,882 unique HLA haplotypes. Each row contains six alleles (two pairs for A, B and C).

Output file (data2.csv):

A∗02:01,A∗02:01,B∗35:05,B∗40:02,C∗03:04,C∗04:01

A∗02:01,A∗24:02,B∗35:05,B∗40:08,C∗03:04,C∗04:01

A∗02:01,A∗24:02,B∗35:05,B∗35:05,C∗04:01,C∗04:01

A∗02:01,A∗68:17,B∗35:19,B∗40:08,C∗03:04,C∗08:01

### Selection of HLA class I pool

**Timing: <1 min**

26. Based on the unique list of HLA haplotypes (21,882), the minimal list of HLA class I alleles covering 98% of the haplotypes should be obtained. Run the following command in the terminal:

python3 ./global_alleles.py data2.csv data3.csv

***Note:*** The output file (**data3.csv**) contains 386 HLA class I. The script allows you to change the threshold (98%) in line 9.

Output file (data3.csv):

A∗01:01

A∗01:02

A∗02:01

### Determination of HLA class I ligands by netMHCpan-4.1

**Timing: 15 min**

This step predicts the interaction of the HLA class I molecules selected in the previous step with filtered peptides obtained after processing.

27. Before inputting peptides into the algorithm, filter them by length, selecting those between 8 and 16 amino acids.

***Note:*** Peptides longer than 8 amino acids are then truncated from the N-terminus to lengths of 8, 9, and 10 amino acids. As a result, you will obtain six groups of peptides: (1) peptides of 8 amino acids; (2) peptides of 9 amino acids; (3) peptides of 8 amino acids derived from 9-amino-acid peptides; (4) peptides of 10 amino acids derived from peptides of 10 or more amino acids; (5) peptides of 9 amino acids derived from peptides of 10 or more amino acids; (6) peptides of 8 amino acids derived from peptides of 10 or more amino acids. The output file contains two columns: truncated peptide and full peptide.

28. Run the following command in the terminal:

./separate_length.sh peptide.csv omicron

***Note:*** This example demonstrates the process of analysis run on peptides derived from the Omicron B.1.1.529 RBD antigen. To run it on peptides derived from the Wuhan-Hu-1 RBD, the second argument should be replaced with ‘wt’ instead of ‘omicron’. ERAAP (ERAP1) cleaves broad spectrum of N-terminal amino acids in epitope precursors, but efficiency of the truncation may significantly differ.^6^ Additionally, cytosolic aminopeptidases, acting before ERAAP, such as thimet oligopeptidase (TOP) and tripeptidyl peptidase II (TPPII) may contribute to the trimming of the HLA class I peptide ligands.^7^ Thus, our *in silico* truncation of epitope precursors may not ideally reflect complexed intracellular pathway of antigen processing.

Output files (8.length, 8_9.length, 8_10.length, 9.length, 9_10.length, 10.length).

Output file (10.length):

RDISTEIYQA ERDISTEIYQA

YKLPDDFTGC IADYNYKLPDDFTGC

VYAWNRKRIS ASVYAWNRKRIS

FVIRGDEVRQ ADSFVIRGDEVRQ

29. Organize the list of alleles to match the algorithm’s required format. Run the following command in the terminal:

awk -F'∗' '{print "HLA-"$1$2}' data3.csv >data4.csv

***Note:*** The program converts the A∗XX:YY format into HLA-AXX:YY.

Output file (data4.csv):

HLA-A01:01

HLA-A01:02

HLA-A02:01

30. Copy the list of peptides of various lengths and the formatted list of HLA class I into the netMHCpan-4.1 folder. Run the following command in the terminal:

cp run_netMHCpan.sh data4.csv ∗length ./netMHCpan-4.1

31. Execute an algorithm predicting the binding of HLA class I to antigen-derived peptides. Run the following command in the terminal:

cd netMHCpan-4.1

./run_netMHCpan.sh 0.5

***Note:*** As an argument, you will obtain a numerical value representing the binding rank threshold. Peptide-HLA class I pairs with a binding rank above this threshold are removed. By default, the binding rank threshold for strongly binding peptides is 0.5. The program returns a list of HLA class I alleles and the corresponding peptides that exhibit strong binding, with a rank below 0.5 (**data5.csv**).

Output file (data5.csv):

A∗68:23,ADSFVIRGDEVR

A∗68:24,ADSFVIRGDEVR

A∗02:06,ADYNYKLPDDFTGC

A∗02:07,ADYNYKLPDDFTGC

### Population immunity analysis

**Timing: 5 min**

This step calculates the population protection index as the average number of peptide-positive HLA class I alleles in a haplotypes within a country. It considers countries for which information on six alleles (pairs A, B and C) for each individual is available. The number of matching alleles out of the six with the list (**data5.csv**) indicates the protection index of an individual.

32. Copy the file **data5.csv** from the netMHCpan-4.1 folder into Coronavirus folder.
  a. Run the following command in the terminal:

cp data5.csv ../

33. Calculate the population protection index for 27 countries (**data6.csv**) and for each peptide in each country (**data7.csv**).
  a. Run the following command in the terminal:

cd ../

./population_immunity.sh

***Note:*** The resulting table (**data6.csv**) with a list of 27 countries is sorted from the highest to the lowest index value (column 2). The number of countries may vary, depending on the file **table.txt** from the step 17. The resulting table (**data7.csv**) contains three columns: country, index value, and peptide.

Output file (data6.csv):

New Zealand,5.82635

Spain,5.6715

India,5.63465

Bolivia,5.60976

United Arab Emirates,5.59615

Russia,5.5954

Portugal,5.58772

Costa Rica,5.57404

Iran,5.5625

Mexico,5.52304

Sri Lanka,5.50758

Nicaragua,5.50122

Gaza,5.5

Colombia,5.49809

Ireland Northern,5.49162

Hong Kong,5.48075

Guatemala,5.47368

Brazil,5.4697

Chile,5.46667

Malaysia,5.43639

United States,5.43189

England,5.42694

South Africa,5.39547

Netherlands,5.39344

Canada,5.375

Kenya,5.19

Paraguay,4.73333

Output file (data7.csv):

United Arab Emirates,0.192308,ADSFVIRGDEVR

Russia,0.0809883,ADSFVIRGDEVR

Iran,0.046875,ADSFVIRGDEVR

Brazil,0.0900673,ADSFVIRGDEVR

### Check protection index against RBD Omicron with a Telegram bot in a second

**Timing: <1 min**

Do you know that now you can check immune defense against the RBD Omicron variant using a simple Telegram bot? Meet **@Protection_index_bot** – a handy tool that calculates protection index based on HLA class I haplotype in a second!

How it works.

34. Provide a haplotype of interest: submit a list of 6 HLA alleles in a 4-digit code.
35. Get Protection Index: the bot analyzes how many of your HLA class I molecules can present RBD Omicron-specific peptides.
36. Realize immunity strength: the result is a numeric score (0–6) – the higher the number, the more HLA class I alleles are RBD-positive.

***Note:*** This tool is for scientific and informational purposes and does not replace medical advice.

## Expected outcomes

By applying this protocol, researchers may determine key peptides, which may be immunodominant epitopes within the populations of countries for which HLA haplotypes are known. In prior studies, this method successfully demonstrated that mutations in Omicron RBD lead to an increased proteasome-mediated release of certain epitopes, correlating with a potential shift in immunogenicity.^1^ We anticipate that applying this protocol to other viral, self- and neoantigens will provide further insights into autoimmunity, antitumor immunity, viral immune escape and host susceptibility.

## Limitations

The protocol described here has several limitations that users should consider. It is dependent on external tools and datasets, including third-party software such as **netMHCpan-4.1** and the **Allele Frequency Net Database**. Since these resources may undergo updates or changes, reproducibility of the results could be affected. In particular, the HLA haplotype dataset (e.g., ***data.csv***) is time-sensitive, and outcomes may vary if the database is updated or expanded. In addition, the protocol requires access to a **Linux server** equipped with specific software packages such as *gawk* and *tcsh*, as well as sufficient computational resources. Users without root privileges may face difficulties during installation. The protocol also makes assumptions in peptide processing by truncating peptides to 8–10 amino acids for HLA binding predictions, which does not fully account for the complexity of intracellular antigen processing, including the effects of upstream and downstream peptidases such as ERAAP, TOP, or TPPII. Furthermore, the analysis of population immunity relies on a protection index calculated through ***in silico*** predictions, which may not correlate directly with real immune responses at the population level. This analysis is further limited to countries with available HLA data (27 in current protocol), potentially excluding populations with distinct genetic backgrounds. Moreover, in some countries, HLA haplotyping datasets may disproportionately represent closed ethnic communities, leading to distortions in the estimation of actual population coverage. The phenotype of the proteasome itself introduces limitations. Because two symmetrical β rings are present in the proteasome, an immunosubunit β5i can be embedded in either, resulting in isolated immunoproteasomes that may contain a constitutive β5 subunit in one ring. This can influence protein degradation under investigation. Notably, this phenomenon also occurs in natural proteasomes, which may exhibit a mixed immuno-constitutive phenotype.

## Troubleshooting

### Problem 1

Low proteasome yield.

### Potential solution

• Make sure that the cells are effectively selected using puromycin, increase its concentration to 2 μg/mL in DMEM growth medium. [obtaining cell lines stably expressing HTBH-tagged proteasomes].
• Confirm the expression of labeled proteasome subunits via western blotting.
• Increase the number of T75 flasks to two per 20 μl of streptavidin agarose resin. [purification of HTBH-tagged 20S proteasomes].
• Incubate the HTBH-labeled proteasomes bound to the streptavidin-agarose resin after all washings with TEV protease for 16 h instead of 2 h. [purification of HTBH-tagged 20S proteasomes].

### Problem 2

Unwanted proteins elute with proteasomes.

### Potential solution

• Prepare fresh lysates. Avoid using frozen lysates. [purification of HTBH-tagged 20S proteasomes].
• During first two washings incubate the resin in wash buffer 1 for 5 min before centrifugation instead of 2 min, dissociation of the 19S subparticle from the 20S catalytic core should be more effective. [purification of HTBH-tagged 20S proteasomes].
• Repeat resin washing step by centrifugation at 500 g for 3 min with 1 ml of wash buffer 2 two more times. [purification of HTBH-tagged 20S proteasomes].

### Problem 3

Low rate of antigen hydrolysis by proteasome.

### Potential solution

• Preheat the protein at 95°С for 10 min to denature it. [antigen hydrolysis by purified proteasomes].
• Add 0.5 μg of PA28α^8^ to 1.0 μg of purified proteasomes 30 min before antigen hydrolysis reaction. [antigen hydrolysis by purified proteasomes].

### Problem 4

gawk Dependency Issues (Multiple Steps). Required for data processing scripts throughout protocol. Potential Cause: gawk (GNU awk) is not installed on Linux system.

### Potential solution

Check and Install gawk (without root).

# Check if gawk is installed

gawk --version # Should show GNU Awk 5.0+

# Install gawk (with root privileges)

sudo apt install gawk

# Local installation (without root access)

wget https://ftp.gnu.org/gnu/gawk/gawk-5.3.2.tar.gz

tar xvzf gawk-5.3.2.tar.gz

cd gawk-5.3.2/ # Version may vary

./configure --prefix=$HOME/.local

make && make install

export PATH=$HOME/.local/bin:$PATH

### Problem 5

netMHCpan-4.1 Installation Fails (step 18).

#### Potential causes

• Missing dependencies (e.g., tcsh not installed).
• Incorrect folder paths in configuration files.
• Lack of root privileges (if installing without sudo access).

### Potential solution

• Check and install tcsh.

# Check if tcsh is installed

which tcsh || echo "tcsh is not installed"

# Install tcsh (with root privileges)

sudo apt install tcsh

# Local installation (without root access)

wget ftp://ftp.astron.com/pub/tcsh/tcsh-6.24.07.tar.gz

tar xvzf tcsh-6.24.07.tar.gz

cd tcsh-6.24.07

./configure --prefix=$HOME/.local

make && make install

export PATH=$HOME/.local/bin:$PATH

• Modify the netMHCpan script’s shebang to point to the local tcsh path.

### Problem 6

Low Protection Index Variability (Step 25).

Potential Cause: Overly stringent binding rank threshold (default: 0.5) excludes weakly binding peptides.

### Potential solution

• Re-run **run_netMHCpan.sh** with a higher threshold (e.g., 2.0) to include more peptides.

./run_netMHCpan.sh 2.0

## Resource availability

### Lead contact

Further information and requests for resources and reagents should be directed to and will be fulfilled by the lead contact, Alexey A. Belogurov, Jr. (belogurov@ibch.ru).

### Technical contact

Technical questions on executing this protocol should be directed to and will be answered by the technical contact, Maxim T. Ri (maxim.ree@gmail.com).

### Materials availability

This study did not generate new unique reagents.

### Data and code availability

Processing scripts are available at https://github.com/RiMaxim/Coronavirus and https://doi.org/10.5281/zenodo.17157770.

## Acknowledgments

This work was supported by RF project «Processing of viral proteins and autoantigens by multicatalytic proteinase complexes» (A.A.K.). The funders had no role in study design, data collection and interpretation, or the decision to submit the work for publication.

## Author contributions

M.T.R., G.A.S., A.A.K., and A.A.B., Jr., were involved in developing and/or optimizing the methods used in this protocol, as well as in writing and editing the manuscript.

## Declaration of interests

The authors declare no competing interests.

## Supplemental information

Data S1. Sleeping Beauty transposon plasmid pSBi-PSMB4-HTBH-Puro map, related to step 2
